# ApoAI-derived peptide increases glucose tolerance and prevents formation of atherosclerosis in mice

**DOI:** 10.1007/s00125-019-4877-2

**Published:** 2019-05-08

**Authors:** Shelley J. Edmunds, Rebeca Liébana-García, Oktawia Nilsson, Joan Domingo-Espín, Caitriona Grönberg, Karin G. Stenkula, Jens O. Lagerstedt

**Affiliations:** Department of Experimental Medical Science, Biomedical Center Floor C13, Lund University, Tornavagen 10, 221 84 Lund, Sweden

**Keywords:** ApoA-I, Atherosclerosis, Cardiovascular disease, Diabetes, Glucose tolerance test, Glycaemic control, HDL, Insulin, Peptide, RG54

## Abstract

**Aims/hypothesis:**

Finding new treatment alternatives for individuals with diabetes with severe insulin resistance is highly desired. To identify novel mechanisms that improve glucose uptake in skeletal muscle, independently from insulin levels and signalling, we have explored the therapeutic potential of a short peptide sequence, RG54, derived from apolipoprotein A-I (ApoA-I).

**Methods:**

INS-1E rat clonal beta cells, C2C12 rat muscle myotubes and J774 mouse macrophages were used to study the impact of RG54 peptide on glucose-stimulated insulin secretion, glucose uptake and cholesterol efflux, respectively. GTTs were carried out on diet-induced insulin-resistant and *Lepr*^*db*^ diabetic mouse models treated with RG54 peptide, and the impact of RG54 peptide on atherosclerosis was evaluated in *Apoe*^−/−^ mice. Control mice received ApoA-I protein, liraglutide or NaCl.

**Results:**

The synthetic RG54 peptide induced glucose uptake in cultured muscle myotubes by a similar amount as insulin, and also primed pancreatic beta cells for improved glucose-stimulated insulin secretion. The findings were verified in diet-induced insulin-resistant and *Lepr*^*db*^ diabetic mice, jointly confirming the physiological effect. The RG54 peptide also efficiently catalysed cholesterol efflux from macrophages and prevented the formation of atherosclerotic plaques in *Apoe*^−/−^ mice.

**Conclusions/interpretation:**

The RG54 peptide exhibits good prospects for providing glucose control and reducing the risk of cardiovascular disease in individuals with severe insulin resistance.

**Electronic supplementary material:**

The online version of this article (10.1007/s00125-019-4877-2) contains peer-reviewed but unedited supplementary material, which is available to authorised users.

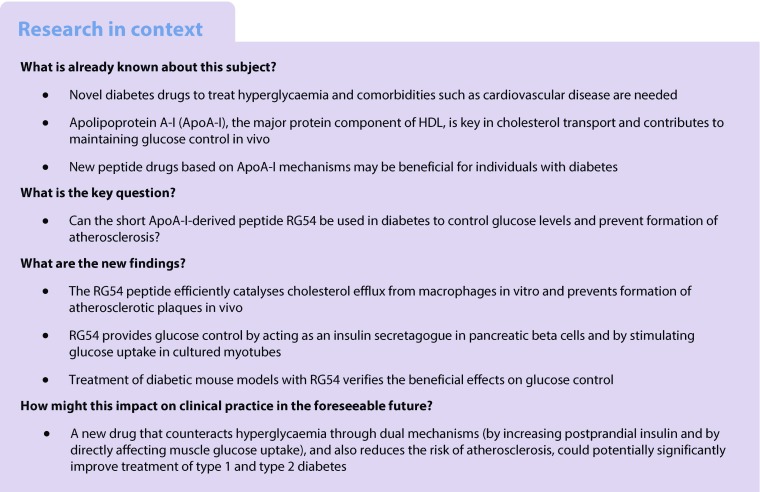



## Introduction

Diabetes treatment has substantially improved in recent years. Yet significant numbers of individuals with diabetes still do not meet their treatment goals or experience undesired side effects. In addition, none of the major diabetes drugs currently in use specifically addresses the inadequate muscular glucose uptake in individuals with insulin-resistant disease. As about 80% of blood glucose in the postprandial state is disposed in skeletal muscle in healthy individuals [1], a lack of this function results in hyperglycaemia and increased risk of muscle fatigue. Individuals with diabetes are also at significantly higher risk of developing chronic complications, including microvascular nephropathy, neuropathy and retinopathy, and macrovascular diabetic foot, as well as cardiovascular disease (CVD) [2, 3]. Novel treatment alternatives that adequately address several aspects of this complex disease are required.

HDL and its major protein constituent apolipoprotein A-I (ApoA-I) have important and well-established functions in the transport and metabolism of cholesterol and other lipids in the circulation, and are considered to prevent atherosclerosis and CVD [4]. ApoA-I has also been implicated in the regulation of glucose control [5, 6], suggesting that ApoA-I may be an important link between diabetes and CVD. Indeed, ApoA-I/HDL stimulates glucose uptake to murine and human cultured myotubes [7–10]. This translates into mouse models of chronic [10] and acute [11] upregulation of human ApoA-I protein that leads to increased glucose-stimulated insulin secretion (GSIS) during GTT, as well as direct stimulation of muscle tissue [12]. Moreover, radiolabelled glucose analogue distribution and positron emission tomography/computed tomography analyses have confirmed muscle as target for increased glucose uptake [12, 13], and also identified the heart as a significant in vivo target [12, 14].

Collectively, ApoA-I/HDL show great promise in novel approaches to treat cardiometabolic diseases. However, several clinical HDL formulations are based on a complex mixture of phospholipids and two ApoA-I proteins per macromolecule. Drugs based on shorter peptides are thus desired. Indeed, in addition to their cholesterol efflux capacity, administration of ApoA-I mimetic peptides to obese mice have been shown to increase insulin sensitivity and improve glucose tolerance [15, 16]. We have previously identified the C-terminal region (54 amino acids; RG54) as the bioactive domain for induction of glucose uptake in vitro and ex vivo [7] and as sufficient for HDL formation [17]. Importantly, the RG54 peptide retains sequence homology with ApoA-I while also adopting a reconstituted (r)HDL-resembling structure in solution [7], thus potentially retaining the many biological functions of the ApoA-I protein in glucose and lipid metabolism. A drug based on the RG54 peptide sequence would therefore hold promise for the treatment of individuals with diabetes and severe insulin resistance. To explore this, we here investigate the RG54 peptide in relevant in vitro and in vivo model systems for its function in insulin secretion, glucose uptake, cholesterol efflux and atherosclerotic plaque formation.

## Methods

### Synthesis, formulation and quality control of synthetic peptides and recombinant proteins

Peptide (lyophilised powder) produced by Red Glead (Lund, Sweden) was reconstituted in Milli-Q water and then either used directly or mixed in a 1:1 ratio with 6 mol/l guanidine HCl in 2× PBS to give a monodisperse solution, followed by desalting using a column equilibrated with PBS, pH 7.4, using ÄKTAxpress fast protein LC (GE Healthcare, Uppsala, Sweden). Peptide integrity was analysed using circular dichroism spectroscopy. Peptide solubility was assayed at Red Glead using HPLC. Peptide stability in physiologic buffer or blood plasma (human or mouse) over 2 h was assayed at the Science for Life Laboratory (Uppsala, Sweden). For detailed methods, please refer to the electronic supplementary material (ESM) Methods.

Recombinant human ApoA-I was produced and purified either in-house or at the Lund University Protein Production Platform (Lund, Sweden) as previously described [7, 18].

### Recombinant HDL formation and lipid clearance

1,2-dimyristoyl-sn-glycero-3-phosphocholine (DMPC; Avanti Polar Lipids, Alabaster, AL, USA) was formed into 100 nm multilamellar vesicles (MLV) via extrusion using the LiposoFast system (Avestin, Ottawa, ON, Canada) as previously described [7]. MLV were incubated with RG54 or ApoA-I at the indicated ratios for 4 days at 24°C to form rHDL. The resulting particle sizes were measured by dynamic light scattering (DLS) using a Zetasizer APS (Malvern Instruments, Uppsala, Sweden).

Turbid MLV solutions at 30 nmol/l DMPC were combined with PBS, RG54 or ApoA-I at a 1:100 molar ratio, and lipid binding at 25°C was measured by absorbance at 325 nm at the indicated times. Readings were fitted to one-way decay of non-linear regression.

### In vitro: study design

Using a range of in vitro models, we assessed the effects of the peptide on HDL formation, cholesterol efflux, insulin secretion and non-insulin-dependent glucose uptake. In vitro experiments were performed in triplicate unless otherwise stated, with each individual experiment regarded as one treatment unit. Peptide effects were measured relative to full-length ApoA-I and vehicle controls. The J774 mouse and C2C12 rat cell lines were sourced from ATCC (Wesel, Germany) and the INS-1E rat cell line was a kind gift from C. Wollheim (University of Geneva/Lund University). All cells were mycoplasma negative. See ESM Methods for more details.

### In vitro: cholesterol efflux assay

The mouse macrophage-like cell line J774 (ATCC TIB-67), loaded with ^3^H-cholesterol, was used for cholesterol efflux to acceptors (RG54 or ApoA-I) as previously described [19].

### In vitro: insulin secretion assay

INS-1E cells were preincubated for 2 h with HEPES KRB supplemented with 3.3 mmol/l glucose and RG54, then stimulated for 1 h with HEPES KRB supplemented with either 3.3 or 20 mmol/l glucose but no RG54 peptide, and insulin secretion was analysed using ELISA (Mercodia, Uppsala, Sweden). See ESM Methods for more details.

### In vitro: glucose uptake measurements

C2C12 myoblasts were differentiated to myotubes by switching from growth media to 2% FBS DMEM for 7 days, with the addition of cytosine arabinoside (Sigma, Stockholm, Sweden) from day 3 to 5 to eliminate proliferating cells [20]. Prior to stimulation, cells starved for 2 h in serum-free DMEM were treated as described and glucose uptake measurements were carried out. See ESM Methods for more details.

### In vivo: animal husbandry and study designs

All animals were housed in conventional shoebox cages, 3–5 animals/cage, maintained in a humidity-controlled room with a 12 h light/dark cycle and had non-restricted food and water except where noted. All animal procedures were approved by the Malmö/Lund Committee for Animal Experiment Ethics, Lund, Sweden. See ESM Methods for more details.

### In vivo: measurement of tissue signalling via Western blotting

Soleus, heart and fat samples were collected from male C57BL/6NTac mice (Taconic, Ejby, Denmark) after 12 h fasting at 9 weeks of age. The effect of RG54 on Akt and AMPK signalling in vivo was then measured by western blot using the following antibodies (all sourced from Cell Signaling Technology/Bionordika, Stockholm, Sweden): pAKT (#4060, 1:2000), Akt (#4691, 1:1000), pAMPK (#2535, 1:1000), AMPK (#2603, 1:1000) and GAPDH (#D16H11, 1:1000). All antibodies were diluted in Tris-buffered saline (154 mmol/l NaCl) with Tween-20 containing 1% BSA and were validated by the manufacturer. See ESM Methods for more details.

### In vivo: atherosclerosis model

Female *Apoe*^−/−^ mice (002052, Jackson Laboratory, Bar Harbor, ME, USA) at 12 weeks of age were acclimatised for 1 week, then changed to the RD western diet (D12079B) and injected i.p. three times per week for 6 weeks with treatments as indicated in ESM Fig. 1. At the end of the experimental period, the mice were euthanised and whole aortas were collected, fixed and stained for analysis. See ESM Methods for more details.

### In vivo: glucose control

Male C57BL/6NTac mice (Taconic, Ejby, Denmark) at 8–9 weeks of age were acclimatised for 1 week on a normal chow diet, then changed to a high-fat diet for 2 weeks (diet-induced obesity; DIO), followed by GTT as indicated. Male *Lepr*^*db*^ (*db/db*) mice on a BKS background (Taconic) at 5 weeks of age were maintained on a normal chow diet and GTTs were performed at 5.5 and 14 weeks of age as indicated. Blood glucose levels were measured in fresh blood (C57: OneTouch Ultra2, Lifescan, Milpitas, CA, USA; *db/db*: GlucoSmart Swing, MSP bodmann, Bobingen, Germany) and insulin levels were assayed in serum using ELISA (Mercodia) at the times indicated in ESM Fig. 2. See ESM Methods for more details.

### Statistics

All numerical data are presented as means ± SEM. Significance was calculated using unpaired, two-tailed Student’s *t* tests or one- or two-way ANOVA with Dunnett’s post hoc multiple comparison using GraphPad Prism (version 8.0, GraphPad Software, San Diego, CA, USA). *p* ≤ 0.05 was considered significant.

## Results

### RG54 peptide displays high solubility/stability and forms rHDL

The peptide RG54 was synthesised with natural amino acids and then modified with an acetylation cap on the N-terminus in order to reduce the susceptibility for proteolytic degradation by endoproteases (ESM Fig. 3a). Reconstituted RG54 peptide, which was shown to have satisfactory solubility (>3 mg/ml), was analysed for homogeneity using DLS. The DLS analyses revealed significant inter- and intra-sample heterogeneity with regard to dynamic size range (ESM Fig. 3b). A chemical denaturation and refolding protocol was therefore employed to successfully generate monodisperse preparations (ESM Fig. 3c). Peptide integrity following the denaturation/refolding procedure was confirmed using gel electrophoresis and circular dichroism spectroscopy analyses (ESM Fig. 3d,e) and by peptide stability assays (buffer or plasma) (ESM Fig. 3f).

Quantitative and qualitative lipid assays were applied to determine the lipid-binding properties of RG54. Solubilisation of MLVs made from DMPC phospholipids by RG54 peptide, or by recombinant ApoA-I protein as control, was assayed by light scattering (ESM Fig. 4a). After 10 min of incubation with MLVs both RG54 and ApoA-I reached similar equilibriums, indicating that the peptide efficiently binds lipids. However, the initial rate of phospholipid binding was significantly lower for the RG54 peptide compared with ApoA-I (ESM Fig. 4a,b).

The reduced initial rate may be due to a more complex binding pattern of the larger number of RG54 peptides needed per formed particle: four to five RG54 molecules (54 amino acids each) are needed to make up the size of full-length ApoA-I (243 amino acids). A qualitative assessment of particle formation was therefore next used to evaluate and compare the sizes of formed rHDL. The hydrodynamic diameters of lipid–protein complexes formed after extended incubation (96 h) of RG54 or ApoA-I with extruded 100 nm MLVs were estimated using DLS. The frequency plots showed that incubation of the MLVs with ApoA-I or RG54 at a 1:100 protein:lipid molar ratio over 4 days resulted in single peaks corresponding to rHDL particles (ESM Fig. 4c,d), whereas incubations with fewer lipids per peptide generated smaller particle sizes (ESM Fig. 4c).

### Peptide RG54 effluxes cholesterol from macrophages in a dose-dependent manner

**Fig. 1:**
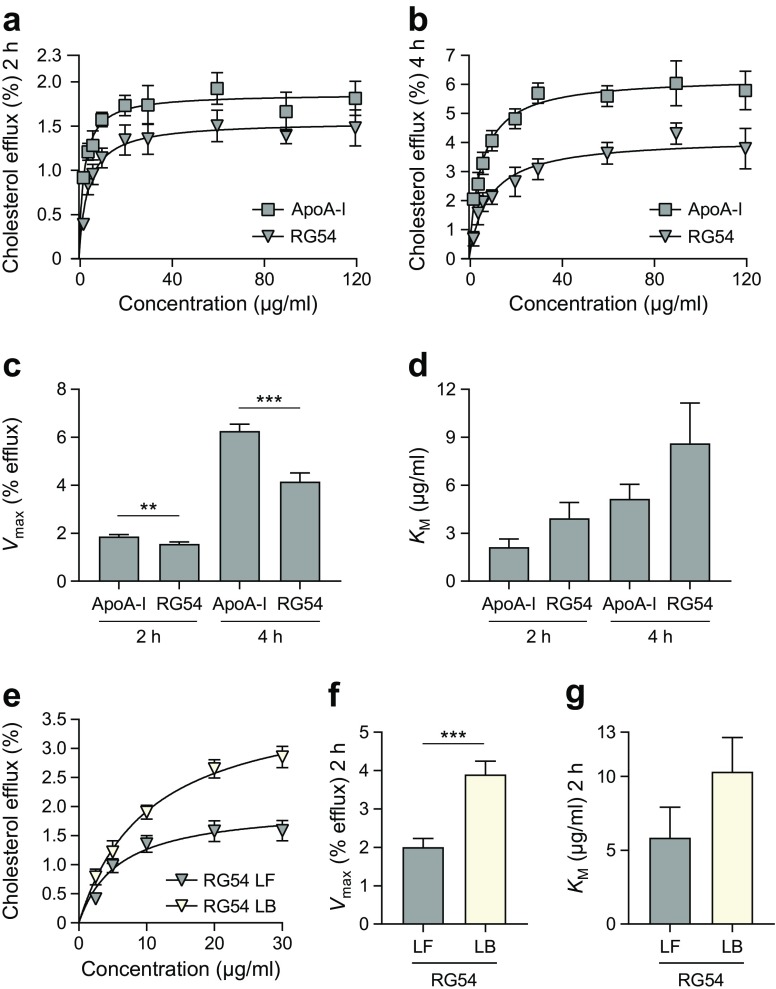
Cholesterol efflux activity of RG54 compared with ApoA-I. (**a**, **b**) Concentration dependence of cholesterol efflux from ^3^H-cholesterol-labelled J774 cells by lipid-free RG54 or ApoA-I was measured after 2 h (**a**) or 4 h (**b**). *n*=5 for both. *V*_max_ (**c**) and *K*_M_ (**d**) were calculated using the Michaelis–Menten equation. (**e**–**g**) Efflux activity of lipid-free (LF) and lipid-bound (LB) RG54 was compared after 2 h. *n*=3. An acyl CoA:cholesterol *O*-acyltransferase (ACAT) inhibitor and 8-(4-chlorophenylthio; CPT)-cAMP were used in all experiments to prevent formation of cholesteryl esters of ^3^H-cholesterol and to induce expression of ABCA1, respectively. Values are means ± SEM. **p≤0.01, ***p≤0.001

To investigate the functionality of the RG54 peptide in a physiological context, cholesterol efflux from J774 macrophages was employed. Cultured macrophages were loaded with radioactive cholesterol, then incubated for 2 or 4 h with RG54 peptide or ApoA-I at the shown concentrations, followed by quantification of the amount of effluxed ^3^H-cholesterol (Fig. 1a,b). RG54 catalysed cholesterol efflux in a dose-dependent manner that followed Michaelis–Menten kinetics, but with a significantly lower *V*_max_ compared with ApoA-I (Fig. 1c). As most circulating ApoA-I is bound to lipids in various species of HDL, and as the RG54 peptide was shown in the lipid clearance assay to efficiently associate with lipids, we reasoned that lipidation of the RG54 peptide might improve its catalytic activity. To explore this, RG54 was first incubated with lipids as described in ESM Fig. 4d, followed by comparative analyses of the catalytic capacities in cholesterol efflux from cholesterol-loaded macrophages. Figure 1e shows that lipidation of RG54 greatly increased the efflux capacity, as shown by a twofold increase in the *V*_max_ value (Fig. 1f).

### Chronic treatment with RG54 reduces early, low-burden atherosclerotic lesion development in *Apoe*^−/−^ mice

**Fig. 2:**
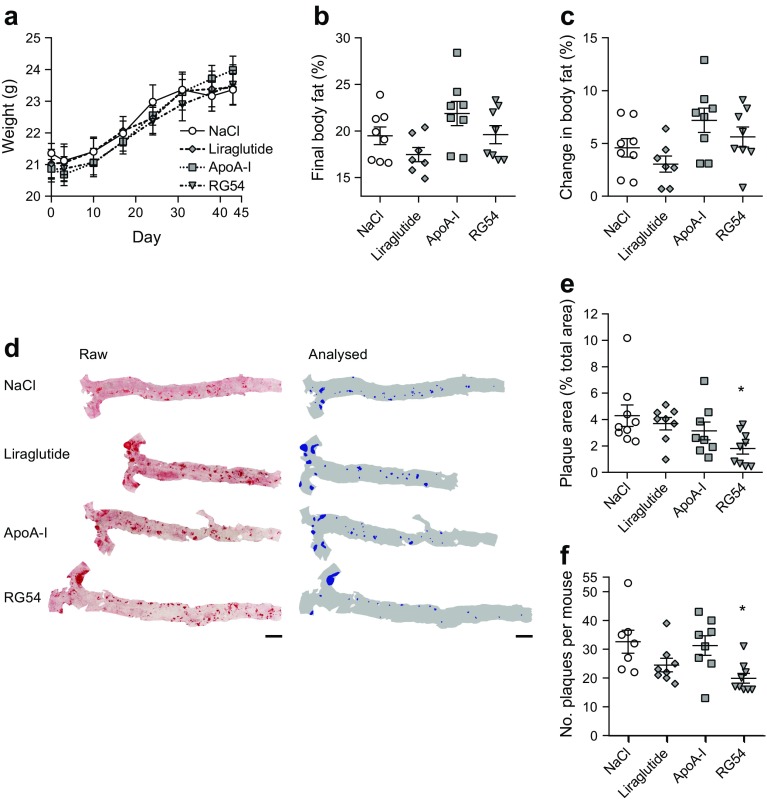
Effect of ApoA-I or RG54 treatment on early, low-burden atherosclerotic lesion development in *Apoe*^−/−^ mice. Female *Apoe*^**−/−**^ mice were placed on a western diet at 12 weeks of age and treated three times per week for 6 weeks with NaCl (200 μl), liraglutide (0.3 mg/kg), ApoA-I (14 mg/kg) or RG54 (12 mg/kg), followed by euthanasia and tissue collection. (**a**) Body weight was measured weekly (*n*=9). (**b**, **c**) Dual-energy x-ray absorptiometry analysis conducted 3 days before treatment commenced and at the end of week 6 is shown as final body fat percentage (**b**) and relative per cent change (**c**) (*n*=8). (**d**–**f**) After euthanasia the aortic arch and abdominal aorta were removed, fixed and prepared en face, then atherosclerotic plaques were stained using Oil Red O. Representative aorta images (**d**) shown as raw images and images analysed for Oil Red O stained areas. Scale bars, 2 mm. Stained areas were then quantified to give the total plaque area (**e**) and total plaques per aorta (**f**) (*n*=9). Values are means ± SEM. *p≤0.05 vs NaCl control

*Apoe*^−/−^ mice, which develop aortic atherosclerosis, were used to test if RG54 reduces the formation of atherosclerotic plaques in vivo. *Apoe*^−/−^ mice were obtained at 12 weeks of age and acclimatised for 1 week, after which baseline body weights, dual-energy x-ray absorptiometry body scans and serum samples were collected. Four groups of nine mice each were then i.p. injected with RG54 (12 mg/kg), ApoA-I (14 mg/kg), liraglutide (0.3 mg/kg), or an equal volume of NaCl solution three times a week for a total of 6 weeks. Atherosclerosis was induced by feeding the mice a western diet with excess cholesterol for the duration of the experimental period. There were no differences in growth curves (Fig. 2a) or body fat composition (Fig. 2b,c) for any treatment group compared with control mice. Serum lipid profiles were measured at the start and end of the experimental period (ESM Fig. 5). We found that, while total cholesterol, HDL and LDL levels in serum increased notably during the experimental period as expected, there were no differences in this increase among treatment groups. Serum triacylglycerol levels increased for all treatment groups during the experimental period except for liraglutide, which showed a modest decrease, giving a significantly different change in triacylglycerol when compared with control mice (ESM Fig. 5d). Serum NEFA also increased by similar levels for all groups during the experimental period.

At the end of the experimental period the abdominal aorta and aortic arch were analysed for atherosclerotic plaque development. All mice showed significant numbers of small and medium plaques along the length of the vessel, indicating early, low-burden atherosclerotic development (Fig. 2d). Quantification of the stained plaques showed that both the number of plaques per mouse and the total plaque area (represented as a percentage of the aortic surface) were significantly reduced in the mice treated with the RG54 peptide but not with liraglutide or ApoA-I (Fig. 2e,f).

### RG54 induces muscle cell glucose uptake and primes INS-1E beta cells to increase GSIS in vitro

**Fig. 3:**
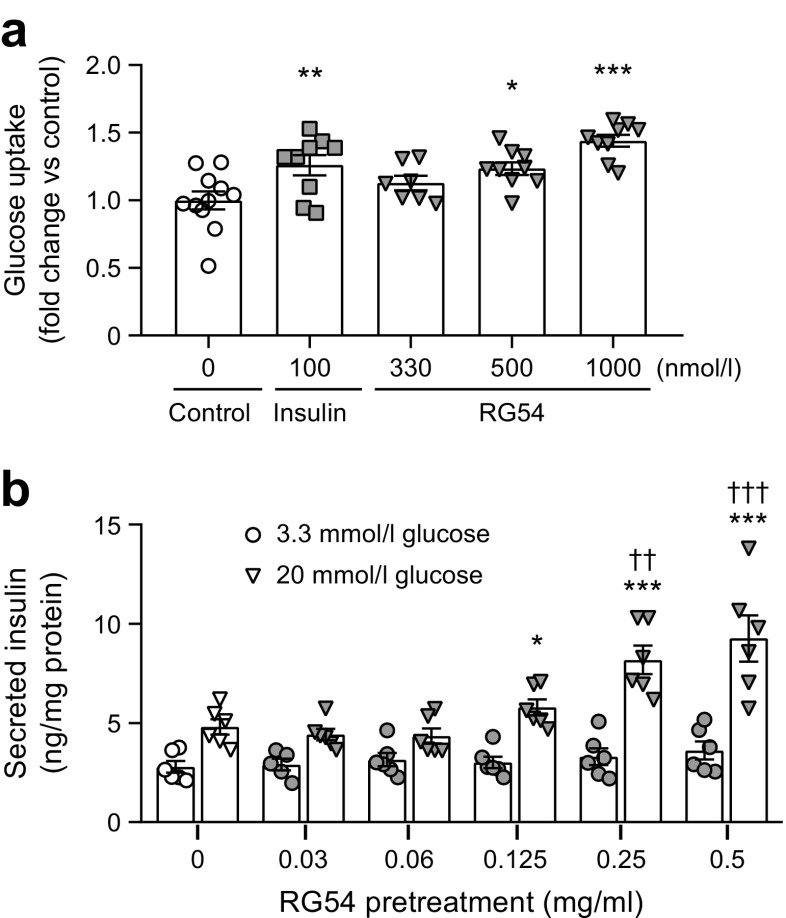
RG54 induces skeletal muscle glucose uptake and primes INS-1E cells to increase GSIS. (**a**) C2C12 skeletal muscle myotubes were incubated in the absence or presence of different concentrations of RG54 or with insulin, followed by glucose uptake measurements. Values are means ± SEM from three independent experiments. *p≤0.05, **p≤0.01, ***p≤0.001 vs control. (**b**) INS-1E cells were incubated for 2 h in the absence or presence of different concentrations of RG54 in a low-glucose environment (preincubation). Buffer was replaced with one containing low (3.3 mmol/l) or high (20 mmol/l) glucose but no RG54 peptide. Insulin secretion was measured after 1 h glucose challenge. Values are means ± SEM from three independent experiments. *p≤0.05, ***p≤0.001 vs 3.3 mmol/l glucose at the same RG54 peptide concentration (preincubation). ^††^*p*≤0.01, ^†††^*p*≤0.001 vs 20 mmol/l glucose with no RG54 peptide in the preincubation

The region of the ApoA-I protein involved in regulating muscle glucose uptake has previously been identified as the C-terminal domain [7] and this was verified here by showing dose-dependent stimulation of glucose uptake by the RG54 peptide (Fig. 3a). However, whether the stimulation of insulin secretion from beta cells by the ApoA-I protein is preserved also in the shorter RG54 peptide is not known, and we therefore investigated this in INS-1E cells. Preincubation with RG54 peptide resulted in dose-dependent increase in GSIS, with up to a twofold increase in GSIS compared with control cells at 20 mmol/l glucose (Fig. 3b). We next explored if the RG54 peptide affected cellular signalling (AMPK and Akt phosphorylation) in soleus muscle, heart muscle and adipose depots in lean mice. However as minor to no changes were observed (ESM Fig. 6), we then focused our studies on preclinical diabetes models.

### Acute treatment with RG54 improves glucose tolerance in preclinical diabetes models

**Fig. 4:**
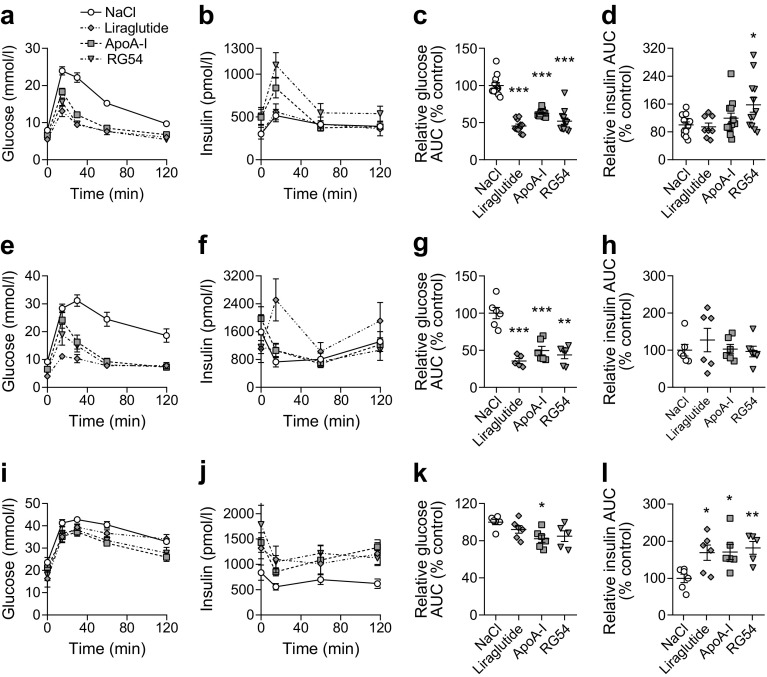
Acute treatment with RG54 improves glucose tolerance during GTTs in three models of type 2 diabetes. Male C57Bl/6 mice fed a high-fat diet for 2 weeks (**a–d**) or *db/db* mice at 5.5 (**e–h**) or 14 (**i–l**) weeks old were fasted overnight, then treated with a single i.p. injection of NaCl (negative control, 200 μl; C57, *n*=12; *db/db* 5.5 weeks, *n*=6; *db/db* 14 weeks, *n*=6), liraglutide (0.1 mg/kg; C57, *n*=11; *db/db* 5.5 weeks, *n*=6; *db/db* 14 weeks, *n*=6), ApoA-I (14 mg/kg; C57, *n*=12; *db/db* 5.5 weeks, *n*=6; *db/db* 14 weeks, *n*=6) or RG54 (12 mg/kg; C57, *n*=12; *db/db* 5.5 weeks, *n*=6; *db/db* 14 weeks, *n*=5). After a further 3 h, mice received an i.p. glucose load (40 mg/mouse for C57; 50 mg/mouse for *db/db*) followed by blood and plasma collection at the indicated times, and blood glucose (**a**,**e**,**i**) and plasma insulin (**b**,**f**,**j**) concentrations were determined. Total AUC was calculated (mmol/l × min for glucose, pmol/l × min for insulin) and then normalised to the average value of the relevant NaCl control group to give relative AUC (% control) for glucose (**c**,**g**,**k**) and insulin (**d**,**h**,**l**). Values are means ± SEM. *p≤0.05, **p≤0.01, ***p≤0.001 vs NaCl control

The effect of RG54 peptide on glucose disposal was investigated in an established insulin-resistant mouse model [11]. DIO mice were fasted for 9 h followed by a single i.p. dose of RG54 (12 mg/kg), ApoA-I (14 mg/kg), liraglutide (0.1 mg/kg), or an equal volume of NaCl solution. After a further 3 h fasting period, GTTs were performed (Fig. 4a,b). Mice receiving RG54, ApoA-I or liraglutide showed a pronounced increase in glucose-clearance capacity (Fig. 4c) compared with mice that had received NaCl, which was accompanied by significantly increased levels of serum insulin for mice treated with RG54 only (Fig. 4d). While 3 h treatment with liraglutide, ApoA-I, or RG54 significantly lowered fasting glucose levels (ESM Fig. 7a), there was no concomitant increase in fasting insulin levels (ESM Fig. 7b) and no change in insulin sensitivity as measured by QUICKI (ESM Fig. 7c).

We next used type 2 diabetes models with a more advanced phenotype: *db/db* mice at 5.5 and 14 weeks of age. At 5.5 weeks of age, control mice showed elevated fasting blood glucose and insulin levels (ESM Fig. 7d) and poor glucose clearance during the GTT (Fig. 4e). Mice receiving RG54, ApoA-I or liraglutide showed a significant increase in glucose-clearance capacity compared with control mice (Fig. 4g). There was a modest, transient increase in insulin secretion at the 15-min time point for mice treated with liraglutide only, but overall insulin AUC was not increased (Fig. 4h). While 3 h treatment with ApoA-I or RG54 significantly lowered fasting glucose levels (ESM Fig. 7d), there were no significant changes in fasting insulin secretion (ESM Fig. 7e), and no concomitant change in insulin sensitivity as measured by QUICKI (ESM Fig. 7f).

At 14 weeks of age, mice were markedly obese with a mean ± SEM body weight of 55.3 ± 0.6 g. Fasting blood glucose of control mice had increased significantly compared with mice at 5.5 weeks of age (mean ± SEM 9.1 ± 0.6 vs 25.5 ± 3.4 mmol/l; ESM Fig. 7d,g). One mouse was excluded from RG54 treatment group because of very high residual insulin secretion prior to the experiment. Mice receiving RG54 or ApoA-I showed an increased glucose-clearance capacity, represented by a lower glucose curve during the GTT (Fig. 4i). However, there was no significant change in AUC for mice treated with RG54 when compared with the NaCl-treated control animals (Fig. 4k), likely due to the reduced group size of *n* = 5. There was a significant increase in insulin AUC for all treatment groups (Fig. 4l), but this was largely due to increased non-GSIS in the 3 h incubation prior to the GTT (Fig. 4j, ESM Fig. 7h).

### RG54 improves glucose tolerance in GTT of DIO mice when administered by i.p. or s.c. injection

**Fig. 5:**
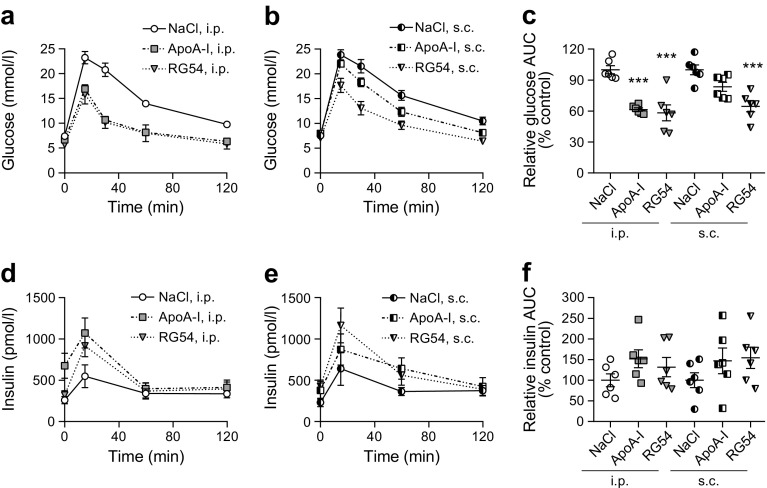
RG54 improves glucose tolerance in GTT in DIO mice when administered by i.p. or s.c. injection, but not when administered by oral gavage. Male C57Bl/6 mice fed a high-fat diet for 2 weeks were fasted overnight, then treated with a single i.p. or s.c. injection of NaCl (negative control, 200 μl, *n*=6), ApoA-I (14 mg/kg, *n*=6) or RG54 (12 mg/kg, *n*=6). After a further 3 h, all mice received an i.p. glucose load (40 mg/mouse) followed by blood and plasma collection at the indicated times, and blood glucose (**a**,**b**) and plasma insulin (**d**,**e**) concentrations were determined for all injection routes. Total AUC was calculated (mmol/l × min for glucose, pmol/l × min for insulin) and then normalised to the average value of the relevant NaCl control group to give relative AUC (% control) for glucose (**c**) and insulin (**f**). Values are means ± SEM. ***p≤0.001 vs NaCl control

**Fig. 6:**
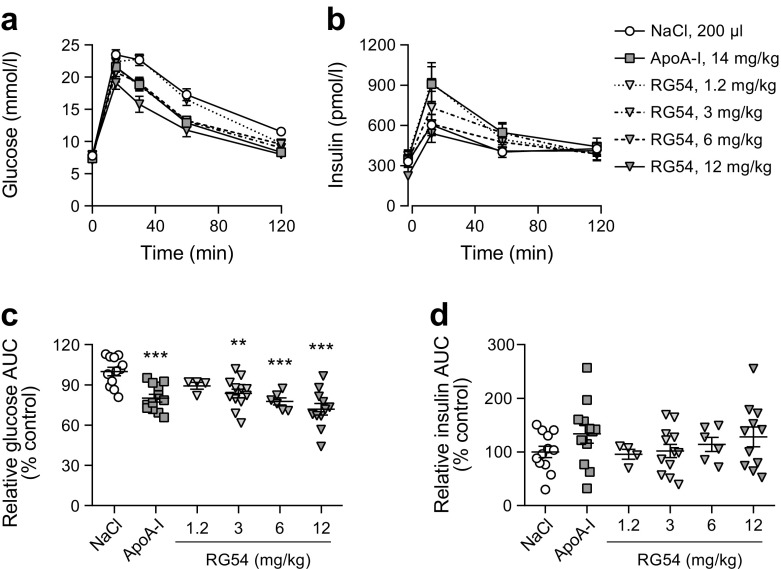
Dose–response analysis of RG54 in improved glucose tolerance during GTT in DIO mice. Male C57Bl/6 mice fed a high-fat diet for 2 weeks were fasted overnight, then treated with a single s.c. injection of NaCl (negative control, 200 μl, *n*=12), ApoA-I (positive control, 14 mg/kg, *n*=12) or RG54 (1.2 mg/kg, *n*=4; 3 mg/kg, *n*=12; 6 mg/kg, *n*=6; 12 mg/kg, *n*=12). After a further 3 h, all mice received an i.p. glucose load (40 mg/mouse) followed by blood and plasma collection at the indicated times, and blood glucose (**a**) and plasma insulin (**b**) concentrations were determined. Total AUC was calculated (mmol/l × min for glucose, pmol/l × min for insulin) and then normalised to the average value of the relevant NaCl control group to give relative AUC (% control) for glucose (**c**) and insulin (**d**). Values are means ± SEM. **p≤0.01, ***p≤0.001 vs NaCl control

We directly compared the i.p. and s.c. injection routes in DIO mice. DIO mice were fasted for 9 h, followed by a single i.p. or s.c. injection of RG54 (12 mg/kg), ApoA-I (14 mg/kg), or an equal volume of NaCl solution. After a further 3 h fasting and incubation period, GTTs were performed. As expected, mice receiving RG54 or ApoA-I by i.p. injection showed a pronounced increase in glucose-clearance capacity and increased insulin secretion compared with control mice (Fig. 5a,d). Similar results were seen when RG54 was injected s.c., with increased glucose clearance and insulin secretion for this group (Fig. 5b,e), and RG54 reduced glucose AUC relative to control by about 40% or 35% in the i.p. and s.c. injected animal groups, respectively (Fig. 5c). Insulin secretion appeared to be slightly increased, but not significantly, for all treatments using both the i.p. and s.c. routes (Fig. 5f). Interestingly, no decrease in fasting glucose or increase in fasting insulin secretion was observed in mice pre-treated via s.c. injection (ESM Fig. 8a,b), despite the improved glucose tolerance provided by the s.c.-injected peptides in the GTT. QUICKI analysis showed no changes in insulin sensitivity (ESM Fig. 8c). Comparison of glucose or insulin curves between the NaCl control groups treated with different injection types demonstrated that treatment route per se did not affect glucose clearance or insulin secretion (ESM Fig. 8d). Finally, we assessed the dose response of s.c. RG54 to find treatment concentrations with full or partial effects on glycaemic control as compared with our positive control (Fig. 6).

## Discussion

The current study investigates the potential of the short RG54 peptide derived from the ApoA-I protein to be used as a treatment in diabetes and cardiometabolic disease via multiple functions.

The RG54 peptide efficiently catalyses cholesterol efflux from macrophages and reduces the formation of atherosclerotic plaques in a rodent atherosclerosis model. Notably, while treatment with the RG54 peptide significantly reduced both aortic plaque area and the number of plaques in atherosclerotic *Apoe*^−/−^ mice, the two comparators, i.e. liraglutide (a glucagon-like peptide 1 receptor agonist) and the ApoA-I protein, failed to prevent atherosclerotic plaque formation in the current study. This finding was unexpected, since earlier studies using experimental animal models have demonstrated that both have anti-atherosclerotic effects. The reason for this may be due to comparably less frequent injections in our study. For example, Gaspari et al described that *Apoe*^−/−^ mice treated twice daily with liraglutide had decreased plaque area and increased plaque stability compared with littermate control animals after 4 weeks of treatment [21], indicating that the dosage of liraglutide in our study was below the cut-off level to achieve significant protection. Similarly, Shah et al convincingly showed that ApoA-I treatment prevented the progression of aortic atherosclerosis in *Apoe*^−/−^ mice treated more frequently and with higher doses of ApoA-I than in the current study [22]. Importantly, since *Apoe*^−/−^ mice treated with the RG54 peptide clearly exhibited reduced levels of plaques despite the relatively few administrations per week, this indicates that additionally improved efficacy can likely be reached by further optimisation of the pharmacokinetics of the RG54 peptide.

Our data also conclude that the RG54 peptide acts on glucose control via dual mechanisms, i.e. by directly stimulating glucose uptake in cultured myotubes and by priming beta cells for improved secretion of insulin following glucose stimulation, which mimics the postprandial state. The in vitro studies are verified by treatments with the RG54 peptide in two rodent diabetes models (DIO and *db/db* mice), which both show improved capabilities to clear blood glucose in the GTTs. Importantly, while the improved glucose tolerance in the DIO mice was accompanied by increased insulin secretion, the RG54 peptide treatment of the *db/db* mice at 5.5 weeks of age led to a significantly improved capability to clear glucose in the GTT without increases in plasma insulin. This finding, which was also true for the animal groups treated in parallel with the ApoA-I and liraglutide comparators, validates the direct and insulin-independent effects of the RG54 peptide on glucose disposal in peripheral tissues. The data also show that s.c. administration is a viable route for the RG54 peptide. This is of relevance, since current diabetes drugs are limited to increasing secretion of endogenous insulin, reducing the reabsorption of glucose in the kidneys, preventing absorption of monosaccharides in the intestine, by lowering liver glucose production and increasing gut energy utilisation, or by directly replacing the endogenous insulin. Drugs based on mechanisms of action that directly stimulate uptake of glucose by skeletal muscle tissues, independent of endogenous and exogenous insulin, are thus needed for individuals who have developed insulin resistance or are experiencing undesired side effects with current treatments. Additional experimental exploration at the cell and tissue level focused on understanding the mechanism of the RG54 peptide would significantly support such ambitions and the design of clinical trials. In order to capture the molecular and cellular impacts, such studies should preferably be performed using metabolically challenged animals, as indicated by our signalling analyses (ESM Fig. 6) and our previous studies on lean animals treated with ApoA-I protein [12].

The increased risk for CVD in diabetes is another challenging problem. Data from large clinical trials focused on CVD outcomes following treatment with glucose-lowering drugs, sodium–glucose co-transporter-2 inhibitors and glucagon-like peptide-1 analogues, have shown significant cardioprotective benefits of these drugs [23, 24]. The biological mechanism that leads to this improved situation for the patient population is not clear. Since the two classes of diabetes drugs both lower blood glucose but through completely different mechanisms, it is plausible that establishing glycaemic control is a strongly contributing factor to the reduced CVD risk, which may involve a reduction in AGEs ([25] and refs therein), potentially including the ApoA-I protein [20, 26, 27]. While only speculative, the finding that the RG54 peptide contributes to glucose control and also prevents atherosclerosis in rodent models suggests that diabetes treatments based on the RG54 peptide may show even greater effects on CVD risk.

The demonstrated biological effects of the RG54 peptide hold promise for the development of a novel diabetes drug with a special focus on treating individuals with moderate to severe insulin resistance. However, the described studies have several limitations, including that the translatability of the biological effects of the RG54 peptide is unclear and has to be tested. A small-scale clinical study performed by Drew et al [8] showed that the reduction of blood glucose in individuals with type 2 diabetes after acute HDL infusion was significantly larger that in placebo control participants, indicating that similar findings on glucose control by HDL as seen cellular and animal model systems are also observed in humans. Another limitation is the administration route. Although many current diabetes drugs are injectables, non-invasive administration is preferred. This is a challenge for a peptide-based treatment, as the peptide will be efficiently hydrolysed in the gut before absorption and entering circulation. However, novel delivery strategies such as inhaled biodegradable calcium-phosphate nanoparticles charged with therapeutic peptides hold promise and may revolutionise the field of peptide-based drugs [28]. In parallel with explorations in oral delivery systems, technological advances in drug pumps already now provide better possibilities for individuals that could also potentially be utilised as a means of administering an optimised RG54 peptide.

This study demonstrates that RG54 peptide improves glucose control and reduces atherosclerotic burden in clinically relevant models, providing a rationale for RG54 peptide as a potential treatment for individuals with type 2 diabetes with poor glucose control and/or at increased risk of CVD, justifying further preclinical evaluation and subsequent clinical testing of this peptide.

## Electronic supplementary material

ESM (PDF 517 kb)

## Data Availability

All relevant data are available in this paper.

## Abbreviations

DMPC: 1,2-Dimyristoyl-sn-glycero-3-phosphocholine
ApoA-I: Apolipoprotein A-I
CVD: Cardiovascular disease
*db/db*: *Lepr* ^*db*^
DIO: Diet-induced obesity
DLS: Dynamic light scattering
GSIS: Glucose-stimulated insulin secretion
MLV: Multilamellar vesicles
rHDL: Reconstituted HDL
