# Supplementary material for: ApoAI-derived peptide increases glucose tolerance and prevents formation of atherosclerosis in mice
Source: Diabetologia. 2019 May 8;62(7):1257–67. doi: 10.1007/s00125-019-4877-2 (PMC6560211; doi:10.1007/s00125-019-4877-2)
Supplement: Supplementary file 1 — (PDF 517 kb) [file 125_2019_4877_MOESM1_ESM.pdf]

Electronic Supplementary Methods

Quality control of synthetic peptides

Peptide concentration was determined at 205 nm using a NanoDrop 2000c spectrophotometer (Thermo Scientific) and integrity was analysed by Tris-Tricine SDS-PAGE followed by coomassie staining or by circular dichroism spectroscopy (CD). CD spectra were acquired on a Jasco J-810 spectropolarimeter equipped with a Jasco CDF-426S Peltier, set to 25°C [1]. Averages of five scans were baseline-subtracted (PBS buffer; 25 mmol/l phosphate, 150 mmol/l NaCl).

Human plasma (citrate) was from Blodcentralen at the Uppsala academic hospital and pooled from four healthy volunteer blood donors after giving informed consent for general research use. Mouse CD-1 plasma (heparin) was from Novakemi AB. Analysis was performed using LC-MS/MS, a Sciex QTRAP 6500 coupled to a Waters Acquity LC system with an attached HSS T3 2 x 50 mm column. A 5%-100% acetonitrile gradient and 0.1% formic acid was used as mobile phase.

In vitro: study design

The primary goal of this study was to investigate the anti-diabetic and anti-atherosclerotic efficacy of a novel ApoA-I derived peptide. Using a range of *in vitro* models, we assessed the effects of the peptide on HDL formation, cholesterol efflux, insulin secretion, and non-insulin dependent glucose uptake. *In vitro* experiments were performed in triplicate unless otherwise stated, with each individual experiment regarded as one treatment unit. Peptide effects were measured relative to full length ApoA-I and vehicle controls.

In vitro: insulin secretion assay

INS-1E rat cells were grown in RPMI-1640 with Glutamax (Invitrogen) supplemented with 10% FBS (Sigma), 1% Penicillin and Streptomycin (Invitrogen), and 50 µM ß-mercaptoethanol (Sigma) and maintained at 37°C and 5% CO2. Cells were plated in 24-well plates at $15x10^4$ cells/well and incubated for 2-3 days prior to the stimulation experiment. Cells were incubated for 2 h with a HEPES Krebs-Ringer bicarbonate buffer (120 mmol/l NaCl, 5 mmol/l KCl, 2.5 mmol/l $CaCl_2$ $2H_2O$, 1.2 mmol/l KH_2PO_4, 1.2 mmol/l $MgSO_4$ $7H_2O$, 25 mmol/l $NaHCO_3$, 10 mmol/l HEPES (pH 7.2) with 0.1% BSA) with 3.3 mmol/l glucose and RG54 as indicated. This was followed by incubation for 1 h with a HEPES Krebs-Ringer bicarbonate buffer with 3.3 or 20 mmol/l glucose as indicated in the absence of the peptide. Supernatants were recovered and insulin secretion was analysed by ELISA (Mercodia). Cells were lysed in RIPA buffer (50 mmol/l Tris-HCl pH 7.4, 1% NP-40, 0.25% Na-deoxycholate, 150 mmol/l NaCl, 1 mmol/l EDTA), total protein concentration measured by BCA assay, and insulin levels normalised to total protein content as a measure of cell density.

***In vitro:* glucose uptake measurements**

C2C12 rat myoblasts (ATCC #CRL-1772) were grown in DMEM (ATCC) supplemented with 10% FBS (Sigma) and 1% Penicillin, Streptomycin and Amphotericin B (Invitrogen) and maintained at 37°C and 5% CO_2. Differentiation to myotubes was achieved by switching from growth media to 2% FBS DMEM for 7 days. From day 3 to 5 in the differentiation process cytosine arabinoside (AraC, Sigma) was added to eliminate proliferating cells [2]. Prior to stimulation, cells starved for 2 h in serum-free-DMEM were treated as described. Glucose uptake measurements were carried out as previously described [3].

***In vivo:* animal husbandry and study designs**

The *in vivo* efficacy studies were intended to gain the data required to justify further testing in future human clinical trials. All animals were housed in conventional shoebox cages with standard wood chip bedding, 3-5 animals/cage, maintained in a humidity-controlled room with a 12-h light/dark cycle, and had non-restricted food and water. Cages were changed twice weekly and food and water refreshed every two days. All peptide or protein treatments were dissolved in sterile 0.9% saline to give injection volumes of 0.1-0.2 mL/mouse, and injected ip or sc as these are the expected injection routes for human usage. Mice were euthanised via cervical dislocation. All animal procedures were approved by the Malmö/Lund Committee for Animal Experiment Ethics, Lund, Sweden.

No statistical methods were used to predetermine sample sizes, but sample sizes are similar to those reported in the field. Each experimental study represented one shipment of mice and control data was compared between shipments where relevant to ensure reproducibility. Mice were age- and sex-matched and mice or samples were randomly assigned to experimental groups or processing orders. Treatments were conducted in the home cages of each mouse and were randomised across cages. Therefore, the experimental unit was individual mice. Group allocation was blinded for all mouse work when possible (such as collection of blood or tissues, sample quantification and analysis, and atherosclerosis scoring). Samples or data points were excluded only in the case of a technical equipment or human error that caused a sample to be poorly controlled.

***In vivo*: measurement of tissue signalling via Western blotting**

Male C57BL/6NTac mice were purchased from Taconic (Ejby, Denmark) at 8 weeks of age and acclimatised for one week on normal chow diet. The mice were fasted overnight, injected ip with RG54 (12 mg/kg, n=4) or NaCl (n=4). All mice were then euthanized 2.5 hours after injection and tissue samples collected from soleus muscle, heart muscle and adipose tissue immediately snap frozen in liquid Nitrogen and stored at -20°C.

Protein was extracted from all tissues by homogenization (Omni TH Tissue Homogenizer, Omni International) in ice cold lysis buffer (50 mmol/l Tris, 270 mmol/l sucrose, 1 mmol/l EDTA, 1 mmol/l EGTA, 5 mmol/l Na-pyrophosphate, 1 mmol/l Na-orthovanadate and 1% NP-40 with protease inhibitors), followed by centrifugation at 13000 g for 10 min at 4°C. The

protein concentrations were analysed with Bradford protein assay. Lysates were heated at 95°C for 5 min in SDS sample buffer containing 10 mmol/l dithiotheritol. Total of 30 µg protein, per lane, was separated by electrophoresis (4-15% Bis-Tris gels, Bio-Rad) and electrotransfered to 0.2 µm nitrocellulose membrane (Bio Rad).

Membranes were blocked for 30 min in 50 mmol/l Tris/HCl pH 7.6, 137 mmol/l NaCl, and 0.1% Tris-buffered saline with Tween-20 containing 5% BSA. The membranes were then probed with antibodies for pAKT (#4060, 1:2000), Akt (#4691, 1:1000), pAMPK (#2535, 1:1000), AMPK (#2603, 1:1000) and GAPDH (#D16H11, 1:1000) (all sourced from Cell Signaling Technology) in Tris-buffered saline with Tween-20 (TBST) containing 1% BSA at 4°C overnight. Detection was performed by HRP-conjugated anti-rabbit IgG secondary antibody (GE Healthcare #NA934V, diluted 1:5000 in 1% BSA in TBST), and chemiluminescence was visualized with LI-COR camera and the Image Studio Software (LI-COR). Ratios of phosphorylated/total protein content were analysed with Image J.

***In vivo*: atherosclerosis model**

Female *Apoe*$^{-/-}$ mice were purchased from The Jackson Laboratory (002052) at 12 weeks of age. Female *Apoe*$^{-/-}$ mice only were chosen for these studies have been shown to more reliably develop significant atherogenic plaques in response to a high cholesterol diet than males [4] thus ensuring the reproducibility of clinical symptoms between animals within the study. After a one-week acclimatisation period, mice were changed to RD Western Diet (D12079B) and injected ip 3 times/week in the early afternoon for six weeks with treatments as indicated (ESM Figure 1). At the start and end of the experimental period all mice were fasted once between 10 am and 2 pm and 0.125 mL blood was collected from the saphenous vein and the following serum lipids were measured according to manufacturer's instructions: HDL-cholesterol (Catalog #79990, Crystal Chem, Downers Grove, Illinois, USA), LDL-cholesterol (Catalog #79980, Crystal Chem, Downers Grove, Illinois, USA), total cholesterol (Infinity Cholesterol Liquid Stable Reagent, Thermo Scientific, Worthing, United Kingdom), triacylglycerides (Infinity Triglycerides Liquid Stable Reagent, Thermo Scientific, Worthing, United Kingdom) and non-esterified fatty acids (ACS-ACOD Method, Wako Life Sciences, Mountain View, California, United States).

Three days later all mice underwent whole-body DEXA scanning. This procedure was performed under isoflurane anaesthesia to ensure adequate mouse immobilization and muscle relaxation during the scan. Mice were weighed weekly during the experimental period. At the end of the experimental period, mice were euthanized in randomised, blinded order between 12 – 2 pm and aortic tissues collected, fixed in Histochoice overnight, then stored in PBS. Aortas were stained with Oil Red O, mounted as flat preparations, and slides were scanned and digitalized using an Aperio ScanScope digital slide scanner (Aperio Technologies, Inc). Slide images were exported and plaque area vs total aortic area was calculated in ImageJ v1.51 for the aortic arch and abdominal aorta. Primary outcomes were size and number of atherosclerotic lesions within the aorta, mouse body weight and composition, and changes in blood lipid profiles.

In vivo: glucose control

Male C57BL/6NTac mice were purchased from Taconic (Ejby, Denmark) at 8-9 weeks of age, acclimatised for one week on normal chow diet, then changed to a High-Fat Diet (HFD; Research Diets D12492, 60% fat content) for 2 weeks, followed by glucose tolerance testing (GTT) with timings as indicated (ESM Figure 2). Male $Lepr^{db}$ mice (*db/db*) on BKS background purchased from Taconic (BKS.Cg-m+/+$Lepr^{db}$/BomTac, Ejby, Denmark) at 5 weeks of age were maintained on normal chow diet and GTTs were performed at 5.5 and 14 weeks of age with timings as indicated (ESM Figure 2). For the GTTs, mice fasted overnight (9 h) were injected with the appropriate treatment, and after an additional 3 h given ip glucose (C57: 40 mg/mouse; *db/db*: 50 mg/mouse) followed by collection of serum samples at the indicated times. Blood glucose levels were measured in fresh blood (C57: OnetouchUltra2, Lifescan; *db/db*: GlucoSmart Swing, MSP bodmann) and insulin levels were assayed in serum using ELISA (Mercodia). *db/db* mice were independently randomized to treatments groups for GTT at each time point as no carry over was expected between experiments due to the acute nature of the treatment effects. Primary outcomes were blood glucose and insulin measurements during the GTT. C57BL/6NTac mice were euthanized immediately after each experiment whereas *db/db* were euthanized immediately after the GTT at 14 weeks of age. Male mice only were chosen for these studies as they have been shown to develop more severe and reproducible clinical symptoms of diabetes than females in these models [5; 6] thus ensuring the reproducibility of clinical symptoms between animals within the study.

ESM Methods References

1. Petrlova J, Dalla-Riva J, Morgelin M, et al.: Secondary Structure Changes in Apoa-I Milano (R173c) Are Not Accompanied by a Decrease in Protein Stability or Solubility. PLoS One 2014;9:e96150

2. Domingo-Espín J, Nilsson O, Bernfur K, Del Giudice R, Lagerstedt JO. Site-specific glycations of apolipoprotein A-I lead to differentiated functional effects on lipid-binding and on glucose metabolism. Biochim Biophys Acta 2018;1864:2822-2834

3. Dalla-Riva J, Stenkula KG, Petrlova J, et al: Discoidal HDL and apoA-I-derived peptides improve glucose uptake in skeletal muscle. J Lipid Res 2013;54:1275-1282

4. Meyrelles SS, Peotta VA, Pereira TM, et al.: Endothelial Dysfunction in the Apolipoprotein E-Deficient Mouse: Insights into the Influence of Diet, Gender and Aging. Lipids in Health and Disease 2011;10:211

5. Pettersson US, Walden TB, Carlsson PO, et al.: Female Mice Are Protected against High-Fat Diet Induced Metabolic Syndrome and Increase the Regulatory T Cell Population in Adipose Tissue. PLoS One 2012;7:e46057

6. Leiter EH, Coleman DL, Hummel KP: The Influence of Genetic Background on the Expression of Mutations at the Diabetes Locus in the Mouse Iii. Effect of H-2 Haplotype and Sex. Diabetes 1981;30:1029-1034

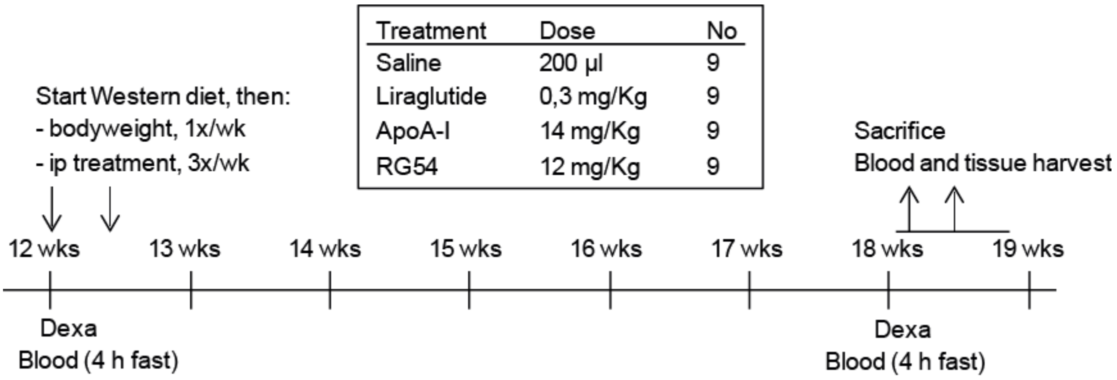

ESM Figure 1. **Scheme of treatment and experimental analysis of atherosclerotic *Apoe*$^{-/-}$ model.**

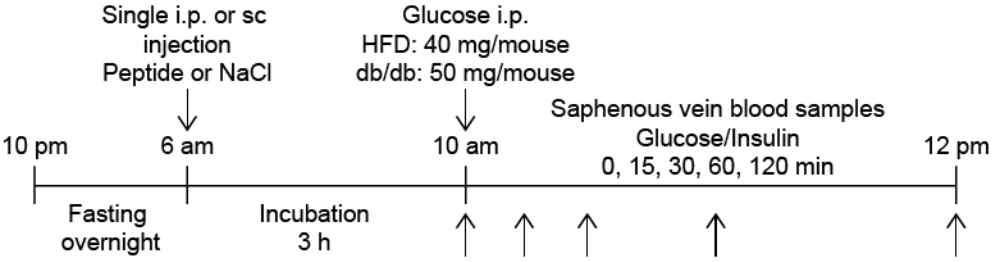

ESM Figure 2. **Scheme of Glucose Tolerance Testing for HFD-fed and *db/db* mice.**

References

a

Peptide	MW (g/mol)	Sequence	Solubility
RG54	6155.98	Ac-AEYHAKATEHLSTLSEKAKPALEDLRQGLLPVLESFKVSFLSALEEYTKKLNTQ-NH2	>3.0 mg/ml

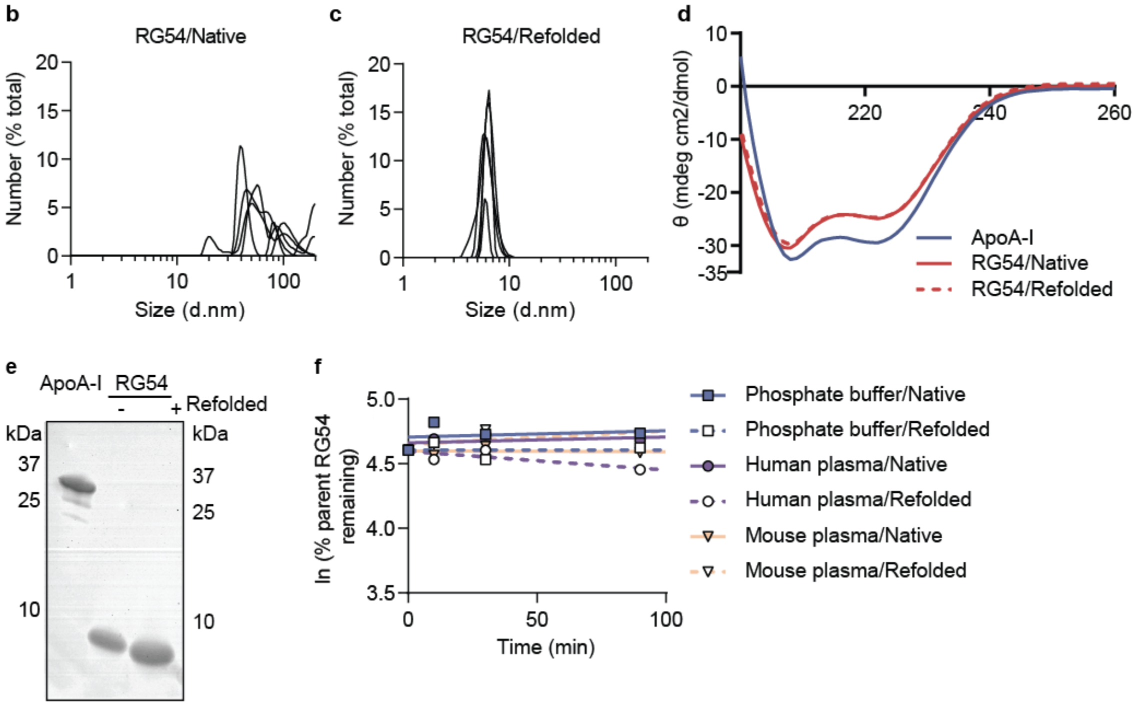

ESM Figure 3. Peptide characterization for native or refolded RG54.

(a) Full peptide sequence. Particle size distribution of RG54 was analysed by DLS and frequency curves generated for Native **(b)** or Refolded **(c)** peptide. Data represents five individual experiments. **(d)** Circular dichroism spectroscopy of RG54 (Native or Refolded) or full length ApoA-I dissolved in PBS at 0.5 mg/mL. This spectrum represents the average of five measurements using five separate solutions. **(e)** Peptide purity as shown by Tris/Tricine SDS PAGE visualized by coomassie staining, 5 mg/well. **(f)** Stability of native or refolded RG54 over time in phosphate buffer, human plasma or mouse plasma.

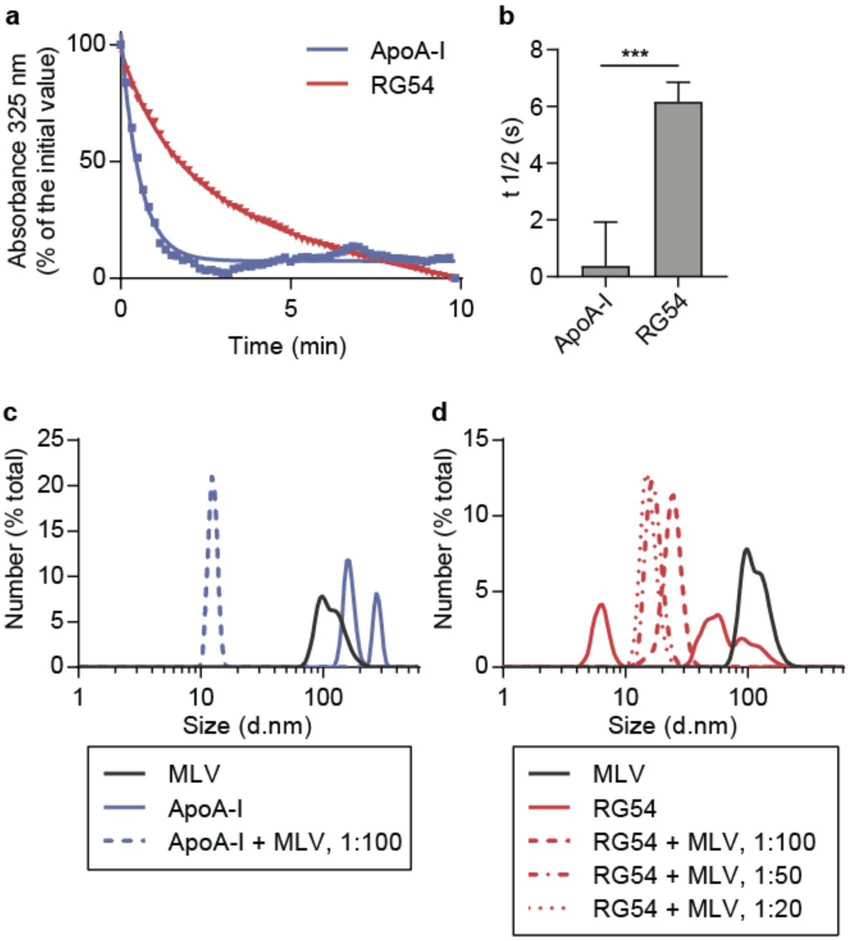

ESM Figure 4. Lipid clearance and peptide-lipid complex formation activity of RG54 compared with full-length ApoA-I.

DMPC clearance activity of RG54 was compared with ApoA-I. Turbid solutions of DMPC MLVs (30 nmol/l) were prepared in PBS as described in Methods and combined with PBS, RG54, or ApoA-I at a 1:100 molar ratio then lipid binding at 25°C was measured by absorbance at 325 nm at indicated times. **(a)** Readings were fitted to one-way decay of non-linear regression. **(b)** The lipid clearance rate is described as t1/2 values calculated from panel a, means ± SEM, n=5, *** $p \leq 0.001$. Further, rHDL particles were prepared by incubating the MLVs with proteins or peptides in the molar ratios noted at 24°C for four days. Particle size distribution was analysed by DLS and frequency curves generated for ApoA-I **(c)** or RG54 **(d)**. Both MLVs alone and lipid-free peptide are included as controls.

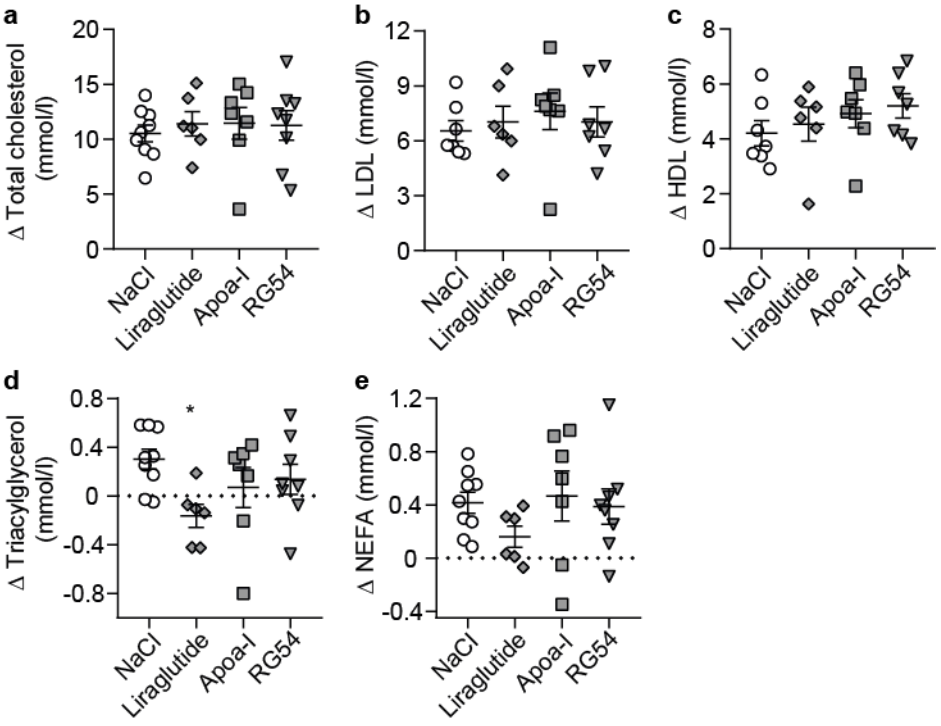

ESM Figure 5. Effect of ApoA-I or RG54 treatment on serum lipid levels in *Apoe*$^{-/-}$ mice. Western diet-fed *Apoe*$^{-/-}$ mice were treated as described in Figure 2, serum lipid levels were measured before and after the treatment period and relative fold change calculated for **(a)** total cholesterol, **(b)** HDL, **(c)** LDL, **(d)** triacylglycerol, and **(e)** non-esterified fatty acids (NEFAs). Values are means ± SEM, * $p \leq 0.05$ vs NaCl control.

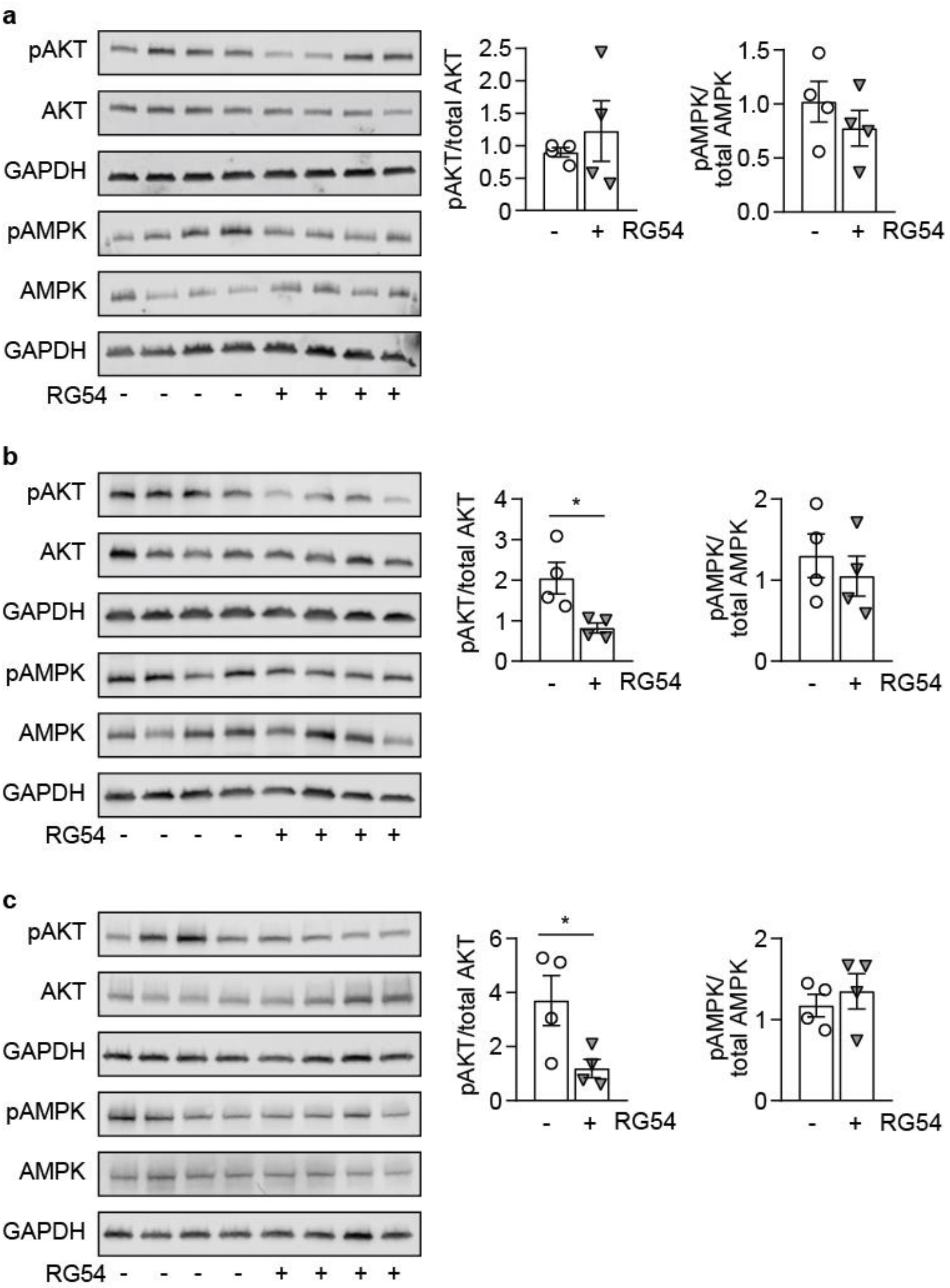

ESM Figure 6. Tissue signalling after treatment with RG54.
Male C57Bl/6 mice at 9 weeks of age were fasted overnight, treated with RG54 (12 mg/kg, n=4) or NaCl (n=4) intraperitoneally, then euthanized after 2.5 hours and tissue samples collected. Proteins were extracted from **(a)** soleus muscle, **(b)** heart muscle, or **(c)** adipose tissue and AKT or AMPK phosphorylation was measured by Western blotting, 30 µg/well. Protein levels were quantified using ImageJ and significance calculated by Student's T-test using GraphPad prism. Values are means ± SEM, * $p \leq 0.05$.

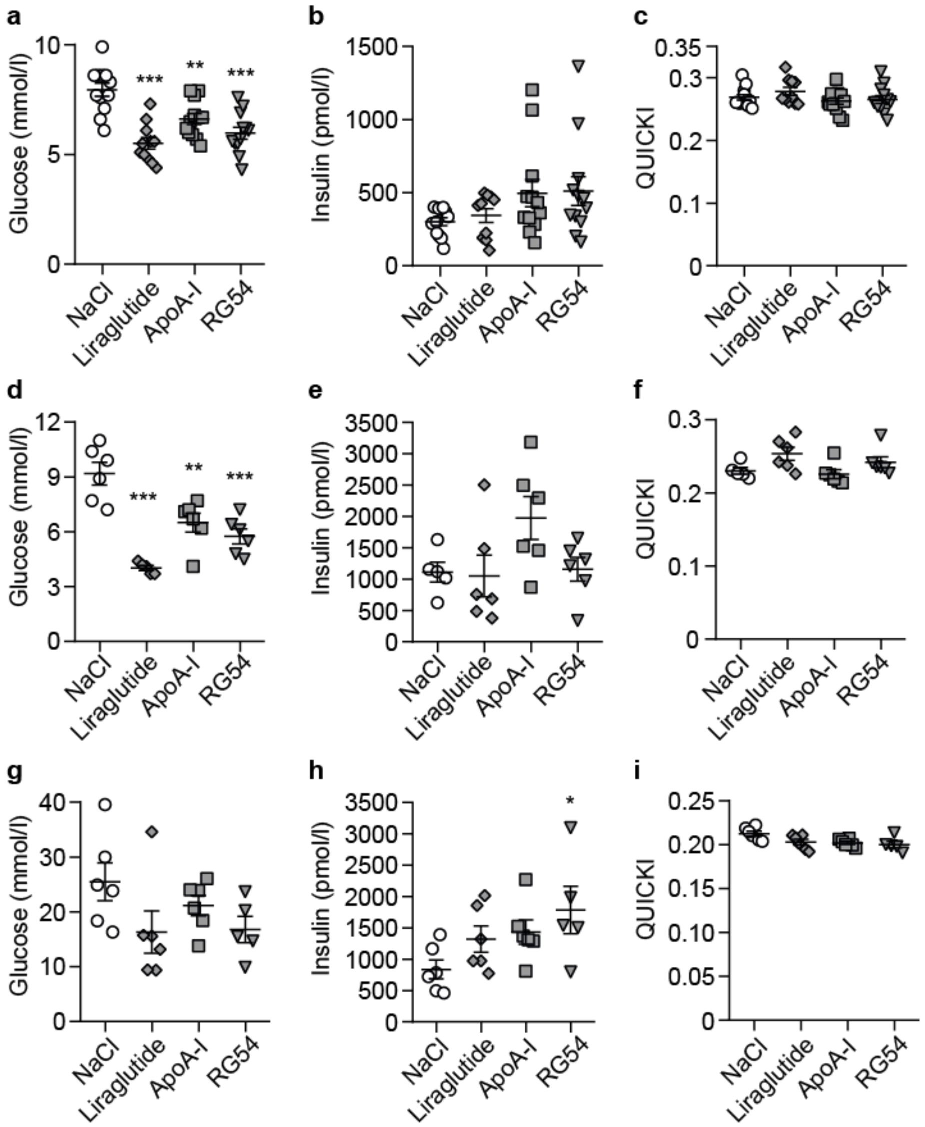

ESM Figure 7. QUICKI analysis after acute treatment with ApoA-I or RG54 in three models of type 2 diabetes.

Male C57Bl/6 mice fed HFD for two weeks **(a, b, c)** or *db/db* mice at 5.5 weeks **(d, e, f)** or 14 weeks old **(g, h, i)** were treated as described in Figure 3. Basal fasting blood glucose **(a, d, g)** and plasma insulin **(b, e, h)** were measured immediately prior to GTT and QUICKI index of insulin sensitivity **(c, f, l)** calculated for all groups. Values are means ± SEM, * $p \leq 0.05$, ** $p \leq 0.01$, *** $p \leq 0.001$ vs NaCl control.

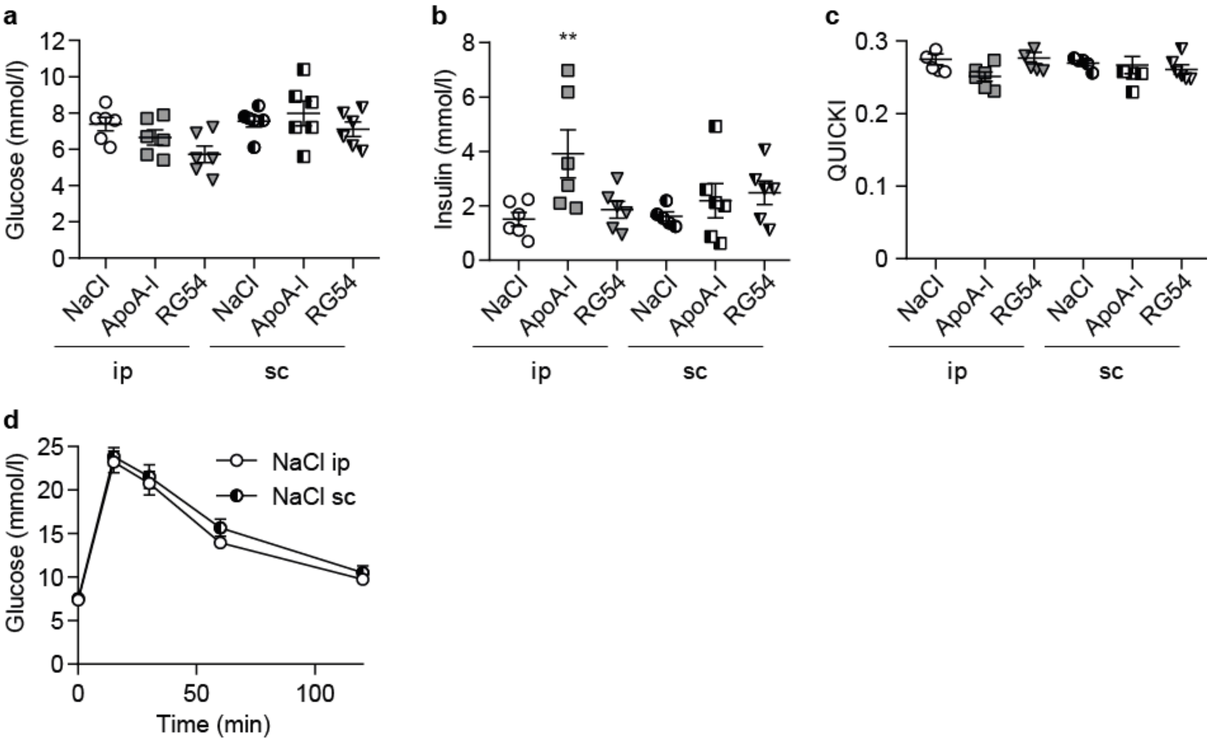

ESM Figure 8. QUICKI analysis of different injection routes for acute treatment with ApoA-I or RG54.

Male C57Bl/6 mice fed HFD for two weeks were fasted overnight, then treated for 3 h with a single intraperitoneal (ip) or subcutaneous (sc) injection as described in Figure 5. Basal fasting blood glucose **(a)** and plasma insulin **(b)** were measured and QUICKI index of insulin sensitivity **(c)** calculated for all groups **(d)**. Blood glucose curves after ip glucose load (40 mg/mouse) were compared for mice treated with NaCl (200 µl/mouse) via ip or sc injections, n=6. Values are means ± SEM, ** $p \leq 0.01$ vs NaCl control.
